# Retinoblastoma Binding Protein 5 Correlates with the Progression in Hepatocellular Carcinoma

**DOI:** 10.1155/2018/1073432

**Published:** 2018-11-07

**Authors:** Huiling Zhou, Jingjing Bao, Xiaowei Zhu, Guihong Dai, Xiaoqin Jiang, Xia Jiao, Haihui Sheng, Junxing Huang, Hong Yu

**Affiliations:** ^1^Department of Pathology, Taizhou People's Hospital, Jiangsu Province, China; ^2^Shanghai Engineering Center for Molecular Medicine, National Engineering Center for Biochip at Shanghai, Shanghai, China; ^3^Department of Oncology, Taizhou People's Hospital, Taizhou, Jiangsu Province, China

## Abstract

Hepatocellular carcinoma (HCC) is one of the most common malignancy tumors with insidious onset, rapid development and metastasis, and poor prognosis. Therefore, it is necessary to understand molecular mechanisms of HCC and identify clinically useful biomarkers for it. This study aimed to investigate the role of retinoblastoma binding protein 5 (RBBP5) in HCC. The expression level of RBBP5 was examined by immunohistochemistry and western blot. The effect of RBBP5 on cell cycle, proliferation, apoptosis, and drug sensitivity was analyzed. RBBP5 was significantly upregulated in HCC tissues and cells. High RBBP5 expression was significantly associated with elevated level of AFP, advanced TNM stage, high Ki-67 expression, larger tumor size, and poor prognosis. Knockdown of RBBP5 significantly inhibited proliferation of HCC cells through cell cycle arrest. In addition, inhibition of RBBP5 increased the sensitivity of HCC cells to doxorubicin. In conclusion, our findings suggest that RBBP5 plays an important role in the progression of HCC and may serve as a novel biomarker and potential therapeutic target for HCC.

## 1. Introduction

Hepatocellular carcinoma (HCC) is the sixth most common cancer and the third most common cause of cancer-related death worldwide with insidious onset, rapid development and metastasis, and poor prognosis [1, 2]. HCC remains a major threat to public health around the world, especially in China. Therefore, the early diagnosis and treatment of HCC is critical to improving patient outcomes. With the progress of molecular biology, it is generally accepted that multiple signaling pathways such as MAPK, PI3K/AKT, and Wnt signaling pathways are the key mechanisms leading to formation of HCC [3, 4]. Since abnormal cell cycle regulation plays an important role in carcinogenesis, investigation of the mechanism of cell cycle regulation may help identify potential biomarkers and therapeutic targets for HCC.

Retinoblastoma binding protein 5 (RBBP5) was determined as the binding protein of RB transcriptional corepressor 1 (RB1) which is one of the best studied tumor suppressor proteins [5]. RBBP5 binds to underphosphorylated pRB1 in the regulation of cell cycle by RB1 pathway [6–8]. RBBP5 has been reported to be involved in a variety of tumors [9]. RBBP5 regulates DNA-damaging agent-induced apoptosis in tumor cells. In glioma, RBBP5 was highly expressed, participated in G1-S transition, and was also associated with the inhibition of apoptosis [10]. Furthermore, RBBP5 is a core member of MLL/SET (mixed lineage leukemia/set-domain containing) complexes involved in tumor cell cycle progression through an MLL–E2F axis which controls the expression of cyclins E, A, and B [11–13]. RBBP5 is required for H3K4 methylation, which is a common marker of transcriptional activity in tumors, such as leukemia [11, 14]. In addition, many other RB1 interacting proteins or their cognate proteins such as SYF2 and Bog are involved in the HCC process [15, 16]. However, the expression and precise role of RBBP5 in HCC remains virtually unknown.

In the present study, we examined the expression level of RBBP5 in HCC and adjacent noncancerous tissues, and the correlation between its expression and clinicopathological parameters. Furthermore, we investigated the effect of knockdown of RBBP5 on the proliferation, cell cycle, apoptosis, colon formation, and drug sensitivity of HCC cells. RBBP5 exhibits potential as a prognostic biomarker and therapeutic target for HCC.

## 2. Materials and Methods

### 2.1. Patients and Tissue Samples

A total of 94 pairs of HCC and paracancerous tissues were obtained from patients who underwent hepatic surgical resection without preoperative systemic chemotherapy at Taizhou People's Hospital between 2007 and 2010. The clinicopathological characteristics of the patients were listed in Table 1. These 94 patients whose average age was 49.0 years (range, 23–74) comprised 76 males and 18 females. In addition, 41.5% of patients were at TNM stages III and IV according to the American Joint Committee on Cancer (AJCC) TNM stage. The follow-up time duration was 5 years, with a range of 1–60 months. Furthermore, additional 8 pairs of fresh HCC and paracancerous tissues were collected for western blot. We obtained the written informed consent from every patient, and the study was approved by the Ethics Committee of Taizhou People's Hospital.

### 2.2. Immunohistochemistry (IHC) and Scoring

The sections were deparaffinized in xylene and rehydrated through graded alcohol. Immunoreactivity was enhanced following antigen retrieved by high temperature and pressure. Endogenous peroxidase activity was blocked with 3% hydrogen peroxide in PBS. After being rinsed in phosphate-buffered saline (PBS, pH 7.2), 10% goat serum was used for 1 h at room temperature to block any nonspecific reactions. The slides were incubated with Anti-RBBP5 antibody (dilution 1:200, Sigma–Aldrich, MO, USA) overnight at 4°C and anti-Ki-67 antibody (dilution 1:500, Millipore, Bedford, MA, USA) at room temperature for 2 h and then incubated with the secondary antibody at room temperature for 1 h. The slides were then counterstained with 3,3′-diaminobenzidine (DAB) and 20% hematoxylin. Finally, the slides were examined under a light microscope (Leica Microsystems, Wetzlar, Germany).

All immunostained slides were assessed in a blinded manner by two pathologists without knowledge of any clinicopathological information. Immunostaining score was calculated for each section according to the proportion of stained tumor cells and the intensity of the staining [17]. The intensity of staining was scored as 0 (no staining), 1 (weakly staining), 2 (moderately staining), or 3 (strongly staining). According to the percentage of positive tumor cells, the extent of staining was scored as 0 (≤ 10%), 1 (11–30%), 2 (31–50%), 3 (51–70%), and 4 (≥ 70%). These two scores were multiplied into a final score (0–12) for each tissue. Samples were classified as low expression (score ≤ 3) or high expression (score > 3).

### 2.3. Western Blot

Cells and tissues were immediately resuspended in a homogenization buffer (50 mM Tris·HCl pH 7.5, 150 mM NaCl, 1% NP-40, 1% sodium deoxycholate, 0.1% SDS, 1 mM EDTA) containing Complete Protease Inhibitor Cocktail (Roche Diagnostics, Mannheim, Germany), and then centrifuged at 12,000 rpm for 30 min at 4°C to collect the supernatant liquid. Total protein concentration was determined using a Bio-Rad protein assay (Bio-Rad, Hercules, CA, USA). The supernatant was diluted in 2× sodium dodecyl sulfate (SDS) loading buffer and boiled for 15 min. Subsequently, the samples were subjected to 10% SDS–polyacrylamide gel electrophoresis (SDS–PAGE) separation and then transferred to polyvinylidene difluoride filter (PVDF) membranes (Millipore, Bedford, MA, USA). After the membranes were blocked with 5% dried skim milk in TBST (20 mM Tris, 150 mM NaCl, 0.05% Tween-20) for 2 h, they were incubated with primary antibodies overnight at 4°C. The membrane was washed with TBST three times for 5 min each, and then horseradish peroxidase-linked IgG (Pierce Biotechnology, Rockford, IL, USA) was added to the membrane as the second antibody at a dilution of 1:5000 according to the manufacturer's instructions. The immune complexes were visualized by chemiluminescence (NEN Life Science Products, Boston, MA, USA).

### 2.4. Cell line and Cell Culture

The human HCC cell lines (Huh7, Hep3B, HepG2, and SMCC-7721) and L02 normal hepatocytes were purchased from Cell Bank of Type Culture Collection of Shanghai Institute of Cell Biology, Chinese Academy of Sciences. (Shanghai, China). All cells were cultured in high-glucose Dulbecco's modified Eagle's medium (DMEM) (Sigma, St. Louis, MO, USA) with 10% fetal bovine serum (FBS) (HyClone, Logan, UT) and 100 U/ml penicillin–streptomycin mixture and in a 37°C incubator with 5% CO_2_.

### 2.5. SiRNA Synthesis and Transfection

Control siRNA and RBBP5 siRNA oligos were synthesized by GenePharma (Suzhou, China). The sequences of RBBP5-targeting siRNA were 5′-GCA AUA CCA CAG CCA UUA ATT UUA AUG GCU GUG GUA UUG CTT-3′ (siRBBP5-3-1); 5′-CCC UGU ACA UCU GGG AGA ATT UUC UCC CAG AUG UAC AGG GTT-3′ (siRBBP5-3-2); 5′-GCA CCA GAC UUC AAA GAA UTT AUU CUU UGA AGU CUG GUG CTT-3′ (siRBBP5-3-3). Cell transfection assays were performed with Lipofectamine 2000 transfection reagent (Invitrogen, CA, USA) according to manufacturer's protocol.

### 2.6. Cell Cycle Analysis

For cell cycle analysis, cells were harvested at a proper time and washed twice with ice-cold PBS and then fixed with 70% ethanol for 24 h at 4°C. Followed by wash with PBS three times, the cells were resuspended in PBS containing RNase A (100 *μ*g/mL) and propidium iodide (100 *μ*g/mL) and then incubated at 37°C for 30 min. Cell cycle distribution was analyzed by BD FACSCalibur flow cytometer (BD Biosciences, CA, USA).

### 2.7. Cell Proliferation Assay

The cells were inoculated at a density of 2 × 10^4^/well into 96-well plate (Corning Inc., Corning NY, USA) and incubated for 24 h. Cell Counting Kit-8 reagents (Dojindo, Kumamoto, Japan) were added to each well at due time, and the cells were incubated for an additional 2 h at 37°C. The absorbance at the wavelength of 490 nm was measured with a microplate reader (Bio-Rad, Hercules, CA, USA).

*Colony Formation Assay*. Cells were inoculated at a density of 200 cells/well into 6-well plates and followed by transfection with control and siRBBP5 transfection according to the manufacturer's instructions. We analyzed the clearly visible colonies (C50 cells/colony) using 0.5% crystal violet stain for 30 min after 14 days of culture.

*Apoptosis Detection*. The cells transfected with siRBBP5 and control cultured for 48 h and were harvested. Apoptosis was assayed using BD FACSCalibur flow cytometer (BD Biosciences, CA, USA) with Annexin V-FITC apoptosis detection kit (Dojindo, Kumamoto, Japan) according to the manufacturer's instructions.

### 2.8. Drug Sensitivity Assay

The cells were inoculated at a density of 2 × 10^4^/well into 96-well plate and incubated for 24 h and then were suspended in DMEM containing 10% (FBS). Cells were treated with different dose of doxorubicin (DOX) 48 hours. The viability of the cells was examined by CCK-8 assay.

### 2.9. Statistical Analyses

All experiments were performed in triplicate. Quantitative variables were expressed as mean ± standard deviation and analyzed using Student's t-test. The* χ*^2^ test was used to analyze the association between RBBP5 and Ki-67 expression and the clinicopathological features. Survival analysis was performed by using the Kaplan–Meier method and the Log-rank test. The Cox's proportional hazards model was used to identify the factors related to prognosis through a multivariate survival analysis. A *P* value < 0.05 was considered statistically significant. All statistical analyses were performed with the SPSS 21.0 statistical analysis software (SPSS Inc., Chicago, IL, USA).

## 3. Results

### 3.1. RBBP5 Was Upregulated in HCC Tissues and Cells

**Figure 1:**
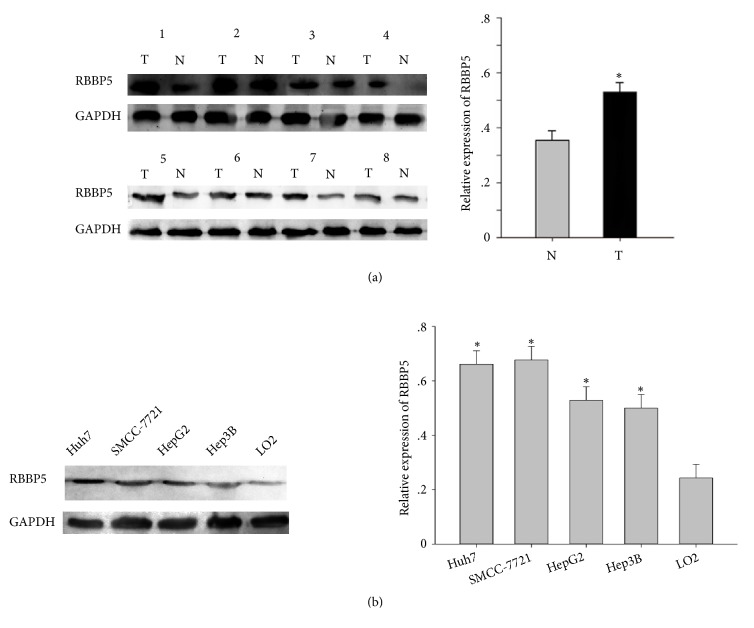
The expression level of RBBP5 in HCC tissues and cell lines. (a) Western blot analysis showed that the expression levels of RBBP5 in HCC tissues (T) were significantly higher than those in adjacent noncancerous tissues (N). (b) RBBP5 expression was upregulated in HCC cells compared with the normal liver cell line (L02). GAPDH was used as a control for protein load and integrity. All experiments were performed in triplicate. *∗P* < 0.05.

To explore the function of RBBP5 in HCC, we first carried out western blot to examine the expression levels of RBBP5 in HCC tissues and cells. The expression levels of RBBP5 in HCC tissues were significantly higher than those in paracancerous tissues (Figure 1(a)). In addition, high expression level of RBBP5 was confirmed in 4 HCC cell lines (Huh7, Hep3B, HepG2, and SMCC-7721) in comparison with a normal hepatocyte cell line (LO2). These findings implicated that RBBP5 was upregulated in HCC tissues and cell lines and might be an important oncogenic factor in HCC.

### 3.2. Clinical Significance of RBBP5 in HCC

**Figure 2:**
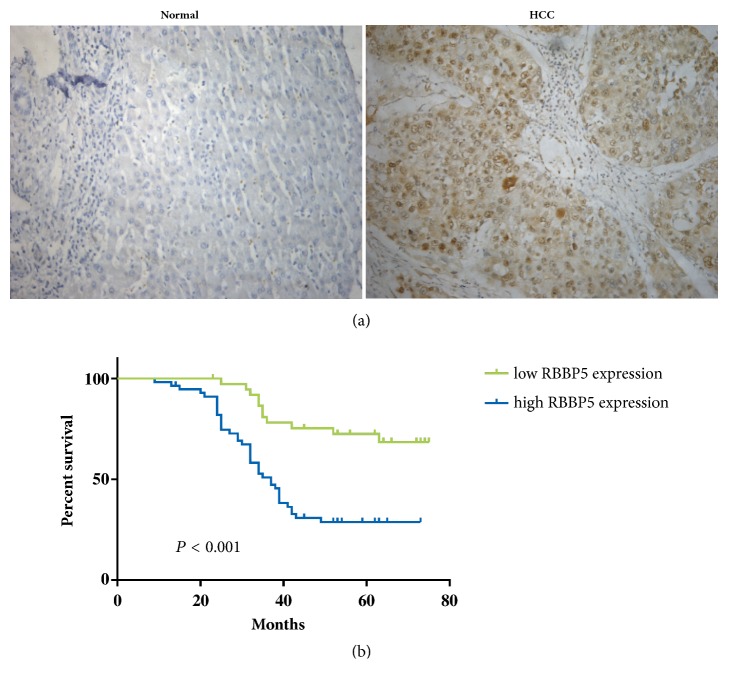
Kaplan–Meier estimates of overall survival for 94 HCC patients according to RBBP5 expression. (a) RBBP5 was significantly overexpressed in HCC tissues (SP× 400) compared with the adjacent noncancerous tissues (SP× 400). All patients were divided into high (n = 56) and low RBBP5 expression groups (n = 38) according to the score. (b) Patients with high RBBP5 expression had shorter survival times than those with low RBBP5 expression (median survival time: 42.9 months* vs.* 63.7 months,* P* < 0.001).

We further investigated RBBP5 expression using IHC in 94 pairs of HCC and paracancerous tissues and evaluated its clinical significance. RBBP5 was significantly upregulated in HCC tissues compared with that in adjacent normal tissues (*P* = 0.013, Figure 2). High RBBP5 expression was significantly correlated with high serum level of AFP (*P* = 0.019), advanced TNM stage (*P* = 0.019), larger tumor size (*P* = 0.012), and high Ki-67 expression (*P* < 0.001) (Table 1). However, no association was observed between RBBP5 expression and other clinicopathological factors, including sex, age, HbsAg, and cirrhosis. In addition, patients with high RBBP5 expression had shorter survival time than those with low RBBP5 expression (*P* < 0.001, Figure 2). Multivariate analysis using the Cox proportional hazards model showed that high RBBP5 expression was an independent factor for prediction of poor outcome in HCC patients (*P* < 0.001, Table 2)

### 3.3. Knockdown of RBBP5 Affects Cell Cycle, Proliferation, and Apoptosis of HCC Cells

**Figure 3:**
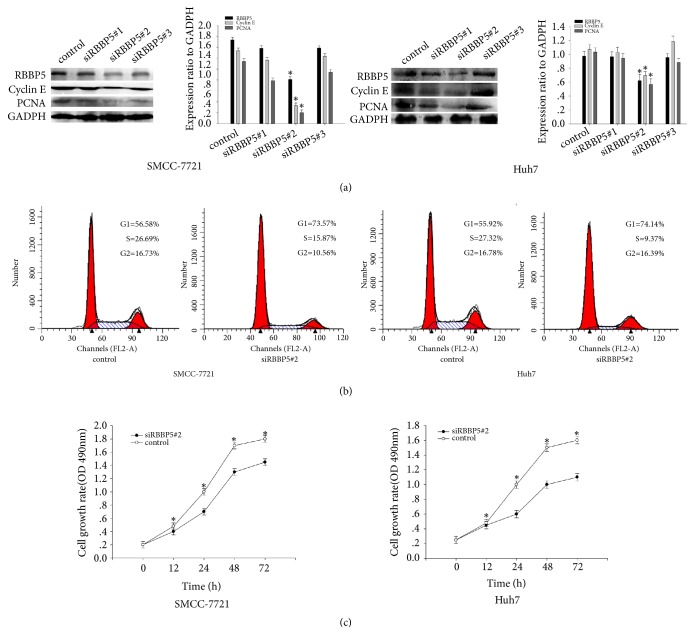
Knockdown of RBBP5 inhibits cell cycle progress and proliferation of HCC cells. (a) Western blot analysis showed that siRBBP5#2 displayed the most significant knockdown effect on RBBP5 expression and were selected for further experiment. After knockdown of RBBP5 by siRBBP5#2, Cyclin E and PCNA were significantly downregulated. (b) Depletion of RBBP5 inhibited cell cycle progression of SMCC-7721 and Huh7 cells. (c) CCK-8 assay showed that knockdown of RBBP5 inhibited proliferation progress of SMCC-7721 and Huh7 cells. All experiments were performed in triplicate. *∗P* < 0.05.

To further investigate the biological function of RBBP5 in HCC, SMCC-7721 and Huh7 cells with the highest level of RBBP5 were selected for further assays. The expression level of RBBP5 was significantly downregulated in SMCC-7721 and Huh7 cells after transfected with siRBBP5 (*P* < 0.05, Figure 3(a)). Not surprisingly, HCC cells were arrested in G1 phase after inhibiting RBBP5 expression (*P* < 0.05, Figure 3(b)). The expression levels of cyclin E and proliferating cell nuclear antigen (PCNA) were obviously decreased after knockdown of RBBP5 (Figure 3(a)). These results indicate that RBBP5 plays an important role in cell cycle regulation. In addition, CCK-8 assays revealed that knockdown of RBBP5 significantly inhibited cell proliferation in SMCC-7721 and Huh7 cells (Figure 3(c)).

### 3.4. Knockdown of RBBP5 Sensitizes HCC Cells to Doxorubicin

**Figure 4:**
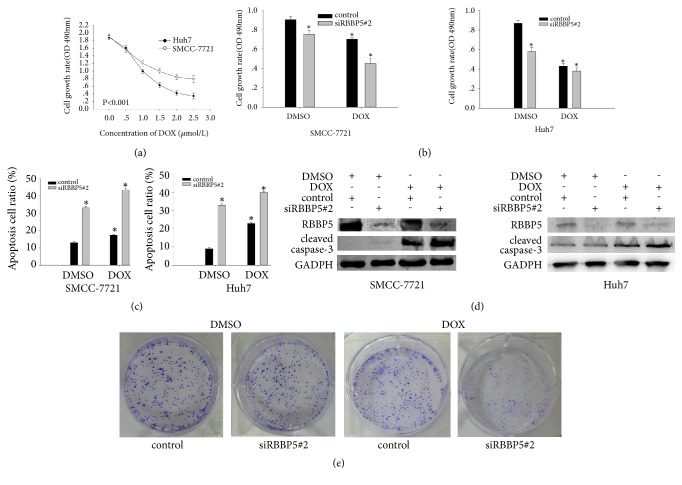
Knockdown of RBBP5 sensitizes HCC cells to doxorubicin. The cell growth of SMCC-7721 and Huh7 cells was influenced by DOX in a dose- and time-dependent manner (a). Cells were treated with DOX (1 mol/L) or not for 48 h. Knockdown of RBBP5 significantly increased DOX-induced cell proliferation inhibition (b), apoptosis (c and d), and colony formation inhibition (e). All experiments were performed in triplicate. *∗P* < 0.05.

DOX affected the growth of HCC cells in a dose- and time-dependent manner (Figure 4(a)). We further examined whether RBBP5 influenced the sensitivity of HCC cells to doxorubicin. SMCC-7721 and Huh7 cells were collected for growth assay and apoptosis analysis after exposure to DOX for 48 h. The growth of HCC cells was decreased, whereas apoptotic rate was increased after knockdown of RBBP5 (Figures 4(b) and 4(c)). Western blot revealed that the level of cleaved caspase-3 was significantly increased after depletion of RBBP5 and the DOX addition (Figure 4(d)). Furthermore, knockdown of RBBP5 significantly suppressed the colony formation of SMCC-7721 and Huh7 cells (Figure 4(e)). These results indicate that inhibition of RBBP5 could increase the sensitivity of HCC cells to DOX.

## 4. Discussion

HCC is a complex disease with high metastasis, recurrence, and chemoresistance despite improvement in HCC diagnosis and therapy [18]. Since its multiple molecular mechanisms have not yet been fully elucidated, the long-term survival of HCC patients is far from unsatisfactory. Therefore, the identification of effective therapeutic targets and biomarkers is a great concern in the field of HCC research. RBBP5, also defined as a binding protein of retinoblastoma, is one of the best studied tumor suppressors [6]. However, the role of RBBP5 in HCC carcinogenesis remains virtually obscure.

Previous studies have shown that RBBP5 is upregulated in some types of human cancers including glioma [10] and multiple myeloma [19]. Overexpression of RBBP5 promotes cell cycle progression and proliferation and induces chemotherapy resistance of cancer cells [10, 19]. In the present study, we found that RBBP5 was significantly upregulated in HCC tissues and cell lines. High RBBP5 expression was associated with aggressive behavior of HCC. RBBP5 was an independent prognostic indicator of survival of HCC patients, which was in agreement with previous study that glioma patients with high RBBP5 expression had worse prognosis [10]. Knockdown of RBBP5 induces cell cycle at G1/S phase and apoptosis and inhibits proliferation in HCC cells. The process may be regulated by direct or indirect stimulation of PCNA and Cyclins. Low RBBP5 expression inhibits cell cycle progression in the process of HCC cell proliferation. Furthermore, inhibition of RBBP5 expression was found to enhance the sensitivity of HCC cells to DOX. These results indicate that RBBP5 plays an important role in the progression of HCC and may be a potential therapeutic target for HCC. However, Liu et al. [19] reported that downregulation of RBBP5 reduced sensitivity to bortezomib and mitoxantrone in RPMI 8226 and NCI-H929 myeloma cell lines adherent to bone marrow stromal cells, indicated that RBBP5 might be the target of bortezomib and mitoxantrone. These results indicate that RBBP5 seems to play different role in the anticancer effect of different drugs.

RBBP5 is also one of the core components of mixed lineage leukemia 1 (MLL1), a histone 3 lysine 4 (H3K4) methyltransferase complex, and is necessary for H3K4 methyltransferase activity[20, 21]. Furthermore, the *β*-propeller domain of RBBP5 has a feature rich surface that can bind nucleic acids and acts as a platform for the recruitment of the MLL complexes to chromatin features or to specific genes [22]. MLL1 is one of the key transcription factors and regulates ~5% of actively transcribed genes [23]. Dysfunction of MLL1 has been reported to be associated with various cancers such as acute leukemia [24, 25]. It has been demonstrated that MLL1 participates in the cell cycle progression [25]. Dysregulation of the cell cycle can lead to uncontrolled cell proliferation and therefore promote tumor development. In this study, we also found that knockdown of RBBP5 inhibited cell cycle and proliferation and promoted apoptosis of HCC cells. These results indicate that RBBP5 may contribute to HCC development by stimulating cell proliferation and inhibiting HCC apoptosis. However, further studies are required to clarify the underlying mechanism in the development and progression of HCC.

In conclusion, our study provided the first evidence that RBBP5 was conspicuously overexpressed in HCC and was associated with Ki-67 expression, AFP, TNM stage, tumor size, and poor prognosis. In addition, knockdown of RBBP5 can suppress the cell cycle and proliferation, induce apoptosis of HCC cells, and increase its sensitivity to DOX. RBBP5 may serve as a novel biomarker and therapeutic target for HCC.

## Figures and Tables

**Table 1:** Correlation of RBBP5 expression with clinicopathological factors in 94 HCC patients.

Clinicopathological factors	RBBP5 expression		*P* Value
	Low	High	
Sex			
female	10	8	0.185
male	28	48	
Age (years)			
< 45	17	18	0.278
≥ 45	21	38	
HbsAg			
negative	11	13	0.631
positive	27	43	
AFP (ng/ml)			
< 50	21	17	0.019
≥ 50	17	39	
Cirrhosis			
negative	19	22	0.397
positive	19	34	
AJCC stage			
I-II	28	27	0.019
III-IV	10	29	
Tumor size (cm)			
< 5	24	20	0.012
≥ 5	14	36	
No. of tumor nodes			
single	24	24	0.062
multiple	14	32	
Capsular formation			
negative	10	26	0.055
positive	28	30	
Metastasis			
negative	31	47	0.786
positive	7	9	
Vein invasion			
negative	27	37	0.658
positive	11	19	
Ki-67 expression			
low	34	2	< 0.001
high	4	54	

**Table 2:** Univariate and multivariate Cox regression analysis of overall survival in 94 HCC patients.

Clinicopathological factor	Univariate analysis		Multivariate analysis	
	HR (95% CI)	*P* value	HR (95% CI)	*P* value
Sex, male vs female	0.999 (0.485-2.058)	0.999		
Age (years), < 45 vs ≥ 45	1.215 (0.669-2.205)	0.522		
HbsAg, positive vs negative	0.738 (0.403-1.351)	0.324		
AFP (ng/ml), ≥ 50 vs < 50	1.339 (0.751-2.389)	0.322		
Cirrhosis, positive vs negative	1.811 (1.035-3.170)	0.038	1359 (0.705-2.619)	0.360
AJCC stage, III+IV vs I+II	1.793 (1.026-3.132)	0.040	1.326 (0.739-2.379)	0.344
Tumor size (cm), ≥ 5 vs < 5	1.126 (0.644-1.969)	0.676		
No. of tumor nodes, multiple vs single	2.845 (1.563-5.180)	0.001	2.417 (1.202-4.860)	0.013
Capsular formation, positive vs negative	0.730 (0.416-1.282)	0.273		
Metastasis, positive vs negative	1.539 (0.786-3.014)	0.209		
Vein invasion, positive vs negative	1.366 (0.772-2.419)	0.284		
RBBP5 expression, high vs low	3.706 (1.880-7.307)	< 0.001	10.631 (3.089-36.592)	< 0.001
Ki-67 expression, high vs low	2.152 (1.139-4.065)	0.018	4.072 (1.227-13.522)	0.022

## Data Availability

The data used to support the findings of this study are included within the article.
